# Computer-modified paramedian approach technique reduces failures and alleviates pain in lumbar puncture: a prospective comparative study

**DOI:** 10.3389/fmed.2023.1293689

**Published:** 2024-01-22

**Authors:** Yuan-Dong Zhuang, Hai-Shu Xie, Jing Chen, Guo-Hua Wu, Jian-Feng Wu, Chun-Mei Chen

**Affiliations:** ^1^Department of Neurosurgery, Fujian Medical University Union Hospital, Fujian Institute of Neurosurgery, Fuzhou, Fujian, China; ^2^Department of Anesthesiology, Fujian Medical University Union Hospital, Fujian Institute of Neurosurgery, Fuzhou, Fujian, China; ^3^Department of Neurosurgery, Pingtan Comprehensive Experimentation Area Hospital, Pingtan, Fujian, China

**Keywords:** computer-modified paramedian approach technique, lumbar puncture, midline approach technique, failure rate, puncture attempts, pain, complications

## Abstract

**Background:**

The conventional midline approach for lumbar puncture (MAT-LP) has a relatively low success rate of 70%. The paramedian approach can increase the effective puncture area and success rate but lacks standardized guidelines. This study evaluated a computer-modified paramedian approach technique (CMPAT) to optimize lumbar puncture using computational techniques.

**Methods:**

In this prospective study, 120 patients underwent CMPAT-LP (*n* = 60) or MAT-LP (*n* = 60). Puncture failure was defined after 6 attempts. Failure rate, number of attempts, pain score, and complications were compared. Subgroup analysis was conducted for age (≥ 50 years).

**Results:**

No significant demographic differences existed between groups. Failure rates were 3.3% for CMPAT vs. 13.3% for MAT. Puncture attempts averaged 2.0 vs. 3.5 and pain scores were 2.7 vs. 4.1 for CMPAT and MAT, respectively. All outcomes were significantly improved with CMPAT, especially in elderly patients. No significant difference in complications was observed.

**Conclusion:**

Compared to MAT, CMPAT-LP demonstrated lower failure rates, fewer puncture attempts, and less pain, without compromising safety. CMPAT may be superior and should be more widely implemented in clinical practice.

## Highlights

- In previous studies, we developed 3D digital models and utilized computer-based spatial analysis to identify an optimal puncture path that maximizes the effective area on the lumbar dural sac and improves fault tolerance. This novel approach is termed the computer-modified paramedian approach technique (CMPAT).
- Compared with the conventional midline technique (MAT), CMPAT demonstrates significantly lower failure rates, fewer puncture attempts, and less procedural pain. However, no significant difference in safety was observed. The advantages of CMPAT were more prominent in patients aged ≥50 years.
- CMPAT is not recommended for severely obese patients, as increased subcutaneous fat thickness can prolong the needle path and hinder needle manipulation, which may increase puncture failures.
- The puncture point of CMPAT is relatively stable, being 1.2–1.5 cm lateral to the caudal spinous process tip, allowing the needle to avoid anatomical structures like the spinous process.
- CMPAT maximizes the target area on the dural sac and provides a wide range of permissible angles, facilitating needle insertion and adjustment during puncture.

## 1 Introduction

Lumbar puncture (LP) is an essential clinical technique used to diagnose and treat many neurological disorders (1, 2). It is routinely performed across medical specialties including neurosurgery, neurology, anesthesia, critical care, pediatrics, emergency medicine, hematology, and rheumatology. In the United States, the estimated utilization rate of LP in emergency departments was 3‰ in 2010 (3). Given its widespread use, proficiency in LP is a fundamental skill for clinicians. Furthermore, LP serves as the foundation for more advanced procedures like lumbar drains and lumboperitoneal shunts (4, 5). Improving LP success rates can thereby facilitate the development of such novel techniques.

Despite its widespread use, conventional midline technique (MAT) for lumbar puncture often fail with high failure rates up to 30% (6–11). Recent evidence shows considerable variability in reported success rates: Williams et al. (9) documented a 28% failure rate using traditional palpation-guided method; Kim et al. (10) reported only 44.7% first-attempt success among 253 patients; Sprung et al. (11) described 64% first-puncture success in 595 individuals undergoing neuraxial anesthesia. Notably, Rabinowitz et al. (6) revealed merely 45% initial success with midline approach in the elderly, versus 85% with paramedian method.

Potential reasons contributing to technical difficulty and failure risks include but are not limited to inter-operator skill differences, methodological variations (e.g., midline approach technique or paramedian approach technique), and case complexity from diverse patient factors like age, obesity and comorbidities (6–11).

Failed punctures may require repeated attempts at multiple vertebral levels, causing substantial patient discomfort. Moreover, the high failure rates, increased number of attempts, and added risks provoke anxiety among clinicians (12). Apprehension and fear of lumbar puncture procedure are also common among patients. When MAT fails at one vertebral level, clinicians often persist with the same technique at a different level. If MAT continues to be unsuccessful, practitioners may resort to ultrasound or fluoroscopic guidance (7, 13). However, most neurologists and neurosurgeons lack training in these alternative LP approaches.

To overcome the limitations of MAT, anesthesiologists introduced the paramedian approach technique for LP (14). By avoiding the narrow interspinous space, paramedian approach can increase the available puncture area on the dural sac and improve success rates (6). Multiple paramedian approach methods have been proposed based on clinical experience, cadaver studies, and radiographic analysis (6, 14–17). However, there is currently no consensus on the optimal paramedian approach due to a lack of standardization (Table 1) (6, 14–17).

**Table 1:** Several non-uniform paramedian approach technique methods.

Title	Author (Years) & Publication	Needle Entry point		Needle Entry Angle		Notes
		Sagittal	Axial	Sagittal	Axial	
Spinal (Subarachnoid)blockade	Cousins and bridenbaugh’s (2008) neural blockade in clinical anesthesia and pain medicine (14)	Directly opposite the cephalad edge of the spinous process below the selected interspace	1.5 cm lateral to the midline	Slightly cephalad 100° to 105° on the cephalad side	About 15° to 20° with the midline	/
A paramedian approach for epidural block: an anatomic and radiologic description	Boon J. (2003) Reg Anesth Pain Med (16)	/	Just lateral to the dorsal spine perpendicular to the skin	Slightly to allow for more superior direction as it is walked cephalad along the lamina. The change in angle between attempts is not more than 10°	With no inward direction	The technique of loss of resistance is used while advancing the needle over the superior ridge of the lamina to confirm entrance into the epidural space.
The Paramedian Technique: A Superior Initial Approach to Continuous Spinal Anesthesia in the Elderly	Rabinowitz, A. (2007) Anesth Analg (6)	L4-5 interspace	1 cm lateral to the midline	Cephalad trajectory at a 10–15° angle	Medial trajectory at a 10–15° angle from the midline	/
Miller’s anesthesia	Miller, R. D. (2015) Miller’s anesthesia (8th Edition) (15)	L3-4 or L4-5 interspace, cephalad spinous process, 1 cm opposite the cephalad	1 cm lateral to the midline	Cephalad trajectory at a 10–15° angle	Medial trajectory at a 10–15° angle from the midline	/
Modified paramedian versus conventional paramedian technique in the residency training: an observational study	Chen, S. -H. (2020) BMC Med Educ (17)	L3-4 or L4-5 interspace, 0.5 cm opposite the cephalad	0.5 cm lateral to the midline	Perpendicular to the skin	Perpendicular to the skin	/

**Figure 1:**
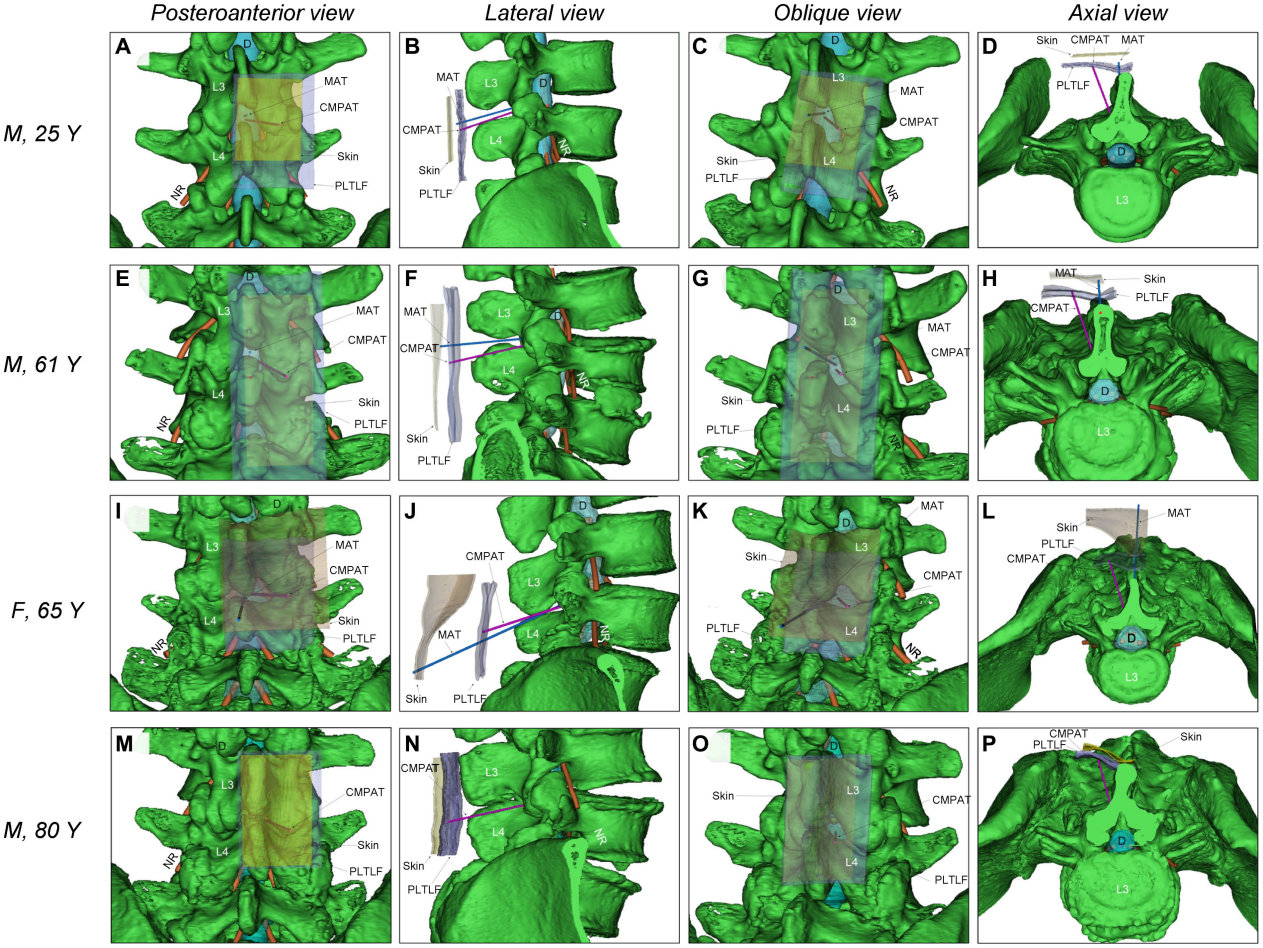
Illustrates the comparison of simulated puncture paths between CMPAT and MAT lumbar puncture. **(A–D)** Demonstrate the simulation of MAT and CMPAT lumbar puncture in a 25-year-old male patient, showcasing the existence of a substantial effective puncture area for both techniques. **(E–H)** Depict the simulation of the lumbar puncture path in an older individual (a 61-year-old male). The MAT simulation reveals interspinous stenosis with limited fault tolerance, whereas the CMPAT simulation exhibits a significant increase in the effective puncture area. **(I–L)** Showcase a 65-year-old female patient with thick subcutaneous fat in the waist. Inaccuracies can easily arise when marking the anchor point on the skin, as seen in other paramedian approach techniques. The space between spinous processes is narrow, and the puncture distance is long, resulting in complications during lumbar puncture with the MAT. **(M–P)** Present an 80-year-old male patient with no space between the interspinous processes. The MAT cannot be successfully simulated, whereas the CMPAT puncture path can be simulated. The increase in effective puncture area using the CMPAT is particularly evident in older patients. CMPAT, computer-modified paramedian approach technique; *D, Dural; MAT, midline approach technique; NR, nerve root; PLTLF, posterior layer of thoracolumbar fascia.

Previously, our team developed a computer-modified paramedian approach technique (CMPAT) for LP based on CT analysis of 90 subjects aged 10–80 years (18). Using thin-slice CT data, we constructed a digital virtual human model comprising the puncture target (dural sac), risk areas (nerve roots), limiting factors (bony structures), and anatomical layers (skin, posterior thoracolumbar fascia) (Figure 1).

**Figure 2:**
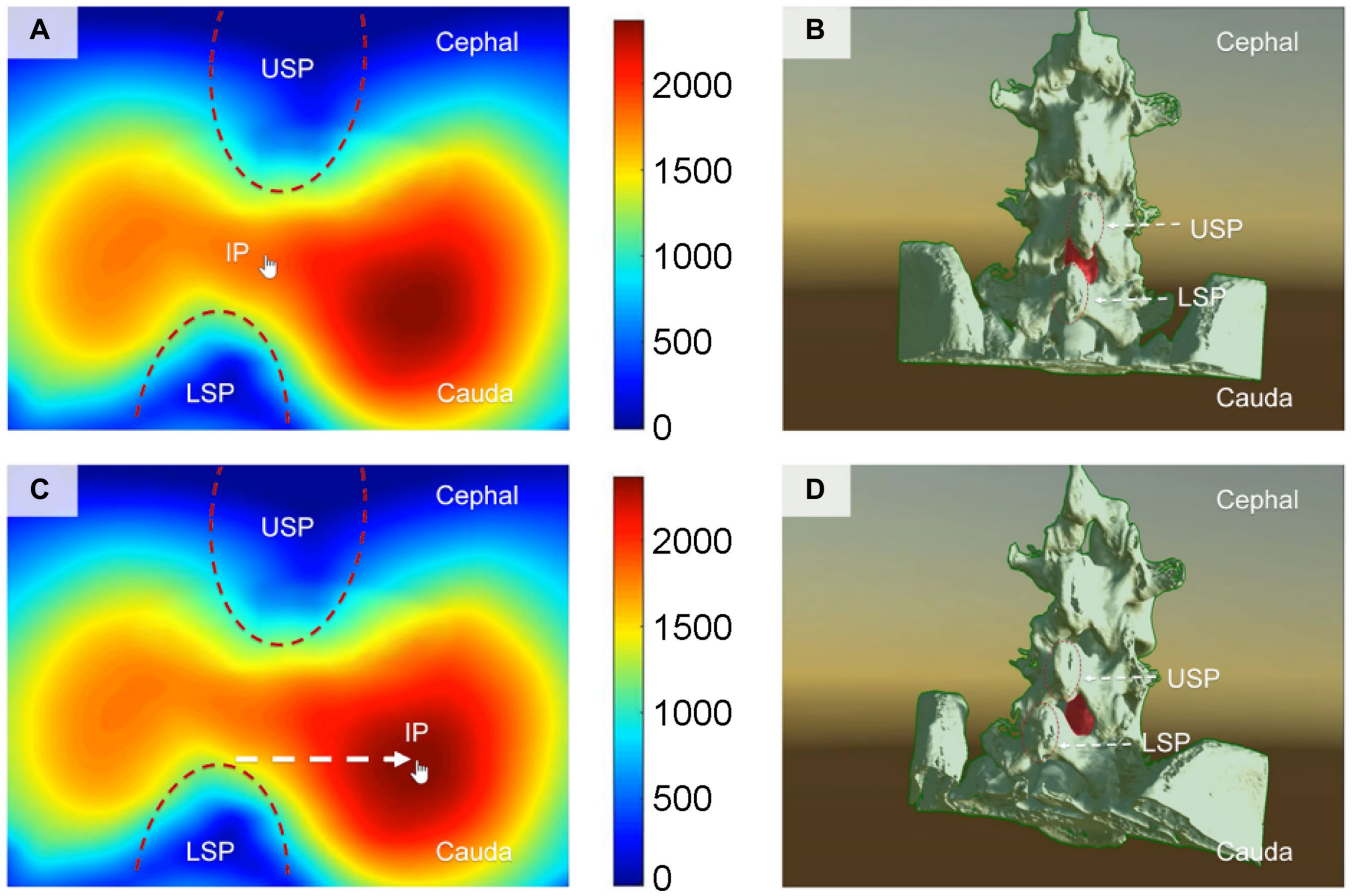
Illustrates the computer-based spatial calculations of the effective area of the lumbar dural sac, which serves as the puncture target, at various insertion points (IPs) for lumbar puncture. **(A,B)** Depict the conventional midline approach technique, while panels **(C,D)** showcase the newly proposed computer-modified paramedian approach technique. The finger arrow indicates the IP. Panels **(A,C)** display the effective area of the puncture target lumbar dural sac calculated using spatial computer-based calculations at different IPs. The cooler color (blue) represents a smaller effective area of puncture, whereas the warmer color (red) indicates a larger effective area of puncture. Panels **(B,D)** present a structural view of the lumbar spine, illustrating the angle (visual angle) of the puncture needle when the finger arrow indicates the IP. The red portion represents the puncture target (lumbar dural sac). The IP for the needle with the largest effective puncture area was found to be 1.2–1.5 cm adjacent to the tip of the lower spinous process (LSP). IP, insertion point; MAT, midline approach technique; CMPAT, computer-modified paramedian approach technique; LSP, lower spinous process; USP, upper spinous process.

**Figure 3:**
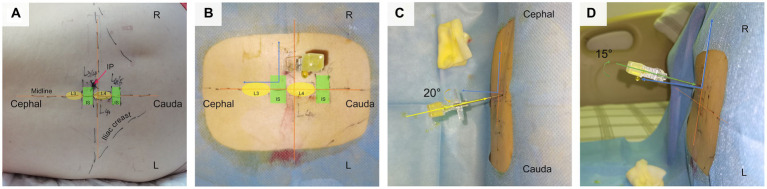
Depicts the application of CMPAT in clinical practice for lumbar puncture. Panel **(A)** illustrates the markings on the skin, indicating the iliac crest, midline, L4 spinous process, and needle insertion point. **(B–D)** Provide different perspectives of CMPAT-LP. CMPAT, computer-modified paramedian approach technique; IP, insertion point; IS, interspinous space.

Through computer simulation of various puncture trajectories, the optimal CMPAT path was identified to maximize the effective puncture area for a wider population range (Figure 2). CMPAT aims to improve LP success rates, reduce needle attempts, minimize pain, and prevent complications (18). The derived CMPAT path allows a specific range of permissible insertion angles (Figure 3), enabling application in the general patient population without needing preoperative CT scans.

While promising, CMPAT requires clinical validation beyond theoretical analysis. Here, we conducted a prospective controlled study comparing CMPAT and conventional MAT for LP, assessing failure rates, number of attempts, pain levels, and complications. To date, no study has applied computational techniques and optimization methodology to plan lumbar puncture paths. This work represents the first effort introducing computer-assisted surgical planning to this field.

## 2 Methods

### 2.1 Clinical data and ethics

This prospective observational study enrolled patients undergoing diagnostic LP at Fujian Medical University Union Hospital from August 2021 to June 2022. The age range was 6–90 years with no BMI restrictions. Patients were assigned to CMPAT-LP (*n* = 60) or conventional MAT-LP (*n* = 60) (6). The study protocol was approved by the hospital ethics committee (No. 2021KJCX019). All patients or legal guardians provided written informed consent. Follow-up data were recorded for 1 day post-procedure by the operator and assigned specialist.

### 2.2 Interventions

**(1) CMPAT group:**

The CMPAT-LP procedure was as follows (Figures 1–3; Supplementary Videos S1, S2).

**Positioning and insertion point:**

The L4 spinous process was first located at the iliac crest’s highest point along the posterior midline. Preference was given to L3-4, with L4-5 as the backup. The lower spinous process in the target interspace served as the key marker. For L3-4, the L4 spinous process was used. The needle insertion point was 1.2–1.5 cm lateral to the spinous tip, allowing avoidance of midline structures while accessing the posterior layer of the thoracolumbar fascia (18).

**Anesthesia:**

Layer-by-layer local anesthesia was administered using 2% lidocaine, focusing on the regions along the planned puncture trajectory.

**Needle insertion:**

The introducer needle was first advanced perpendicular to the skin until reaching the posterior layer of the thoracolumbar fascia resistance. The trajectory was then adjusted, angling the tip 10–30° cephalad in the sagittal plane and 15 ± 5° medially toward the spinal canal midpoint. Gradual advancement continued through the ligamentum flavum until dural puncture and CSF flow were achieved.

If smooth CSF flow through the needle was achieved, the puncture was deemed successful and completed (1). Otherwise, the needle direction was adjusted and a second attempt was made, limiting to a maximum of three attempts. If all three consecutive punctures failed to yield CSF at the L3-4 level, the needle was redirected to the L4-5 interspace for another three attempts (6).

**(2) MAT group:**

Conventional MAT-LP was performed by standard methods as previously described (1).

Both groups were conducted by attending physicians and residents. Puncture failure was determined after 6 unsuccessful attempts, upon which guidance from a senior doctor was sought.

### 2.3 Data collection and outcome measures

Baseline demographics including age and gender distributions were obtained.

The primary outcome was puncture failure, defined as unsuccessful CSF flow after 6 attempts.

Secondary outcomes included the number of puncture attempts, pain scores assessed by a 0–10 numeric rating scale (NRS) (19), and procedure-related complications (e.g., lower limb pain, infection, CSF leakage, cauda equina syndrome, epidural hematoma, and cerebral hernia) (12).

### 2.4 Statistical analyses

Categorical variables were analyzed by chi-squared test. Continuous variables were presented as mean ± standard deviation and compared between groups using independent sample *t*-tests. Pearson correlation examined the relationship between number of attempts and pain scores. Statistical significance was defined as *p* < 0.05. Subgroup analysis was performed for age (≥50 years vs. <50 years). Analyses were conducted using SPSS 23.0.

## 3 Results

### 3.1 Baseline characteristics

**Figure 4:**
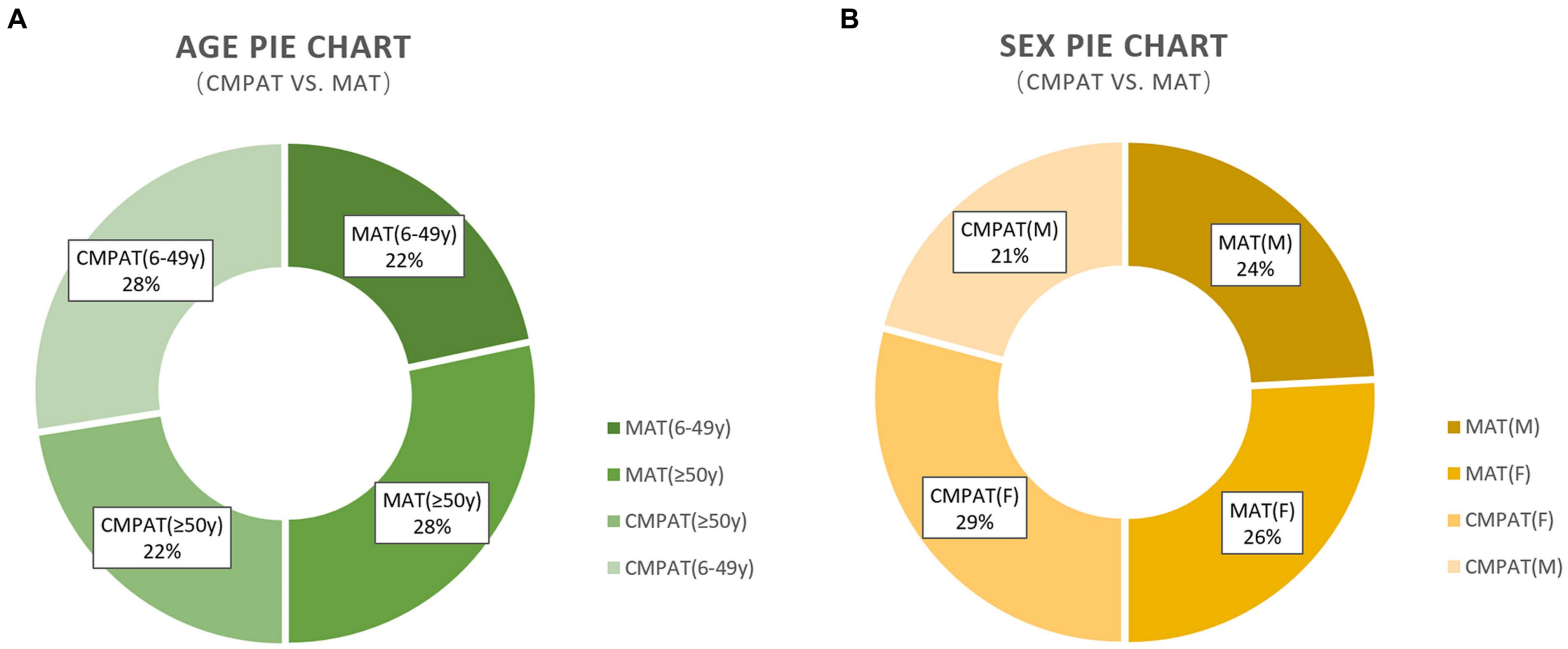
Presents the demographic characteristics, including age and sex, of the study participants. **(A)** Presents the demographic characteristics, including age and sex, of the study participants. **(B)** Demonstrates that there are no differences in the sex composition between the two groups. The numbers displayed in the pie chart represent the count and percentage of individuals included in the analysis. CMPAT, computer-modified paramedian approach technique; MAT, midline approach technique; M, male; F, female.

There were no significant between-group differences in age or gender distribution (Table 2; Figure 4). All participants completed the follow-up.

**Table 2:** Baseline characteristics.

	CMPAT (*n* = 60)	MAT (*n* = 60)	*p*-value
Age (years)			0.201 (>0.05)
6–49	33	26	
≥50	27	34	
Sex			0.463 (>0.05)
Male	25	29	
Female	35	31	

CMPAT, computer-modified paramedian approach technique; MAT, midline approach technique.

### 3.2 Comparison of CMPAT & MAT lumbar puncture results

**Table 3:** Comparison of CMPAT & MAT lumbar punctures.

	CMPAT (*n* = 60)	MAT (*n* = 60)	*p*-value
Failure rate	2/60 (3.3%)	8/60 (13.3%)	0.048 (<0.05)
6–49 y (*n* = 59)	2/33 (6.1%)	2/26 (7.7%)	0.805 (>0.05)
≥50 y (*n* = 61)	0/27 (0%)	6/34 (17.6%)	0.022 (<0.05)
Number of attempts (times)	2.0 ± 1.2	3.5 ± 1.6	0.000 (<0.05)
6–49 y (*n* = 59)	1.8 ± 1.4	2.4 ± 1.5	0.144 (>0.05)
≥50 y (*n* = 61)	2.3 ± 1.0	4.4 ± 1.1	0.000 (<0.05)
Pain NRS	2.7 ± 1.2	4.1 ± 1.9	0.000 (<0.05)
6–49 y (*n* = 59)	2.3 ± 1.3	3.5 ± 2.0	0.013 (<0.05)
≥50 y (*n* = 61)	3.2 ± 0.9	4.6 ± 1.7	0.000 (<0.05)
Unilateral lower limb pain	2/60 (3.3%)	5/60 (8.3%)	0.243 (>0.05)

Data are expressed as mean ± standard deviation or number (percentage). CMPAT, computer-modified paramedian approach technique; MAT, midline approach technique; NRS, numeric rating scale.

**Figure 5:**
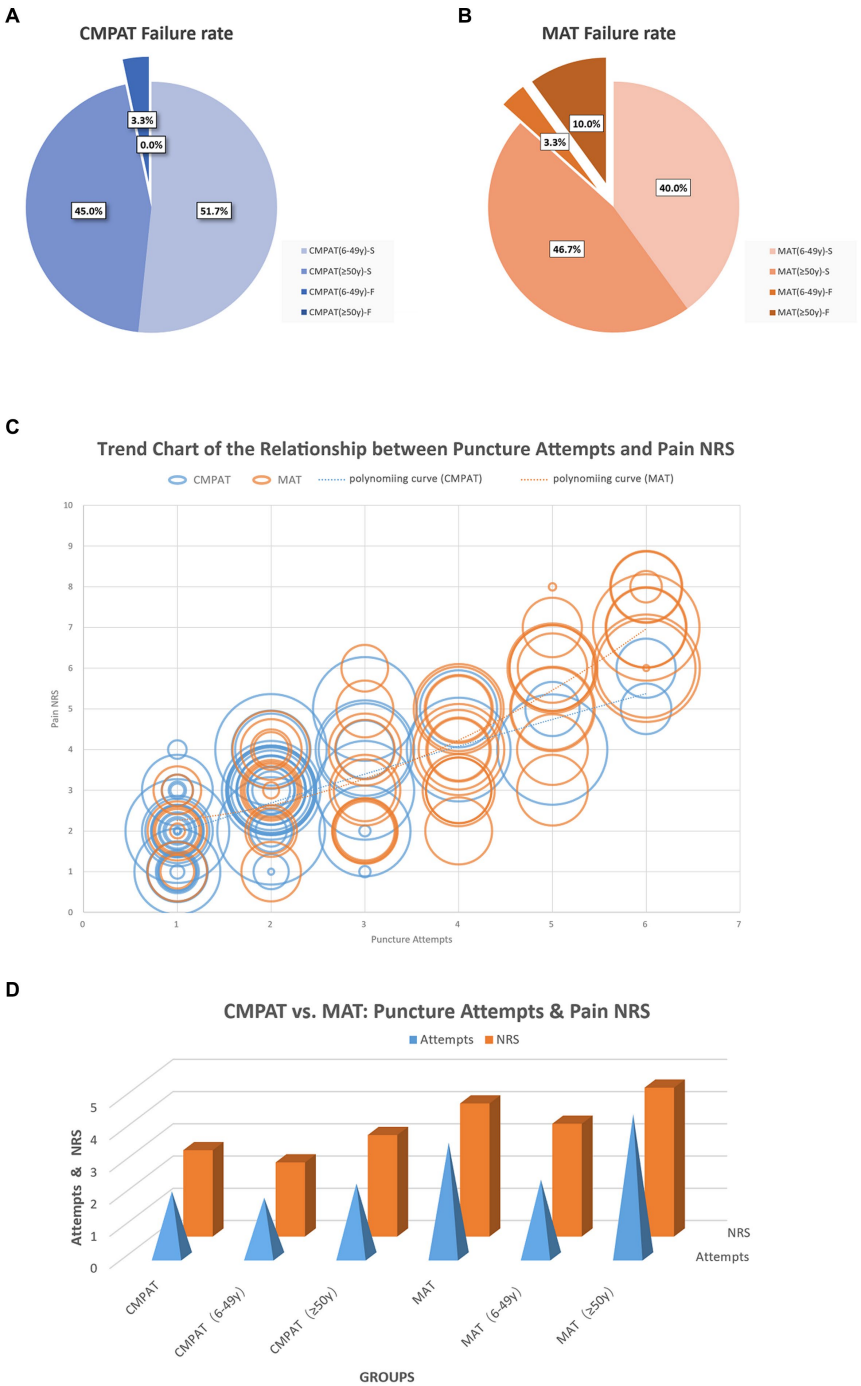
Displays the comparison results between the CMPAT and MAT techniques. Panels **(A,B)** present the failure rates, with a rate of 3.3% in the CMPAT group and 13.3% in the MAT group. Notably, the MAT group shows a higher failure rate in patients aged ≥50 years. Panel **(C)** depicts the relationship between the number of puncture attempts and pain scores. Each circle represents a patient, with the circle’s diameter indicating the patient’s age. The lines illustrate the trend of puncture attempts and pain scores, measured using the numeric rating scale. Patients in the CMPAT group are represented by blue lines and circles, while those in the MAT group are shown with red lines and circles. Pain scores increase with more puncture attempts, but the CMPAT group consistently exhibits lower pain scores compared to the MAT group for the same number of attempts. Panel **(D)** further compares the number of puncture attempts and pain scores between CMPAT and MAT, where red bars indicate pain scores and blue pyramids represent puncture attempts. The CMPAT group demonstrates fewer attempts and lower pain scores than the MAT group. Notably, patients aged ≥50 years in the MAT group exhibit the highest number of puncture attempts and pain scores. CMPAT, computer-modified paramedian approach technique; MAT, midline approach technique; NRS, numeric rating scale; F, failed; S, succeed.

**(1) Failure rate**

The overall failure rate was significantly lower in the CMPAT group (3.3%, 2/60) compared to the MAT group (13.3%, 8/60) (*p* < 0.05) (Table 3; Figure 5).

In the subgroup aged 6–49 years, the failure rates were not significantly different between CMPAT (6.1%, 2/33) and MAT (7.7%, 2/26) (*p* > 0.05).

However, in the subgroup aged ≥50 years, the CMPAT group had a significantly lower failure rate (0%, 0/27) than the MAT group (17.6%, 6/34) (*p* < 0.05).

**(2) Number of puncture attempts**

The overall number of puncture attempts was significantly fewer in the CMPAT group (2.0 ± 1.2) compared to the MAT group (3.5 ± 1.6) (*p* < 0.05) (Table 3; Figure 5).

In patients aged 6–49 years, there was no significant difference in number of attempts between CMPAT (1.8 ± 1.4) and MAT (2.4 ± 1.5) (*p* > 0.05).

In patients aged ≥50 years, the number of attempts was significantly lower in CMPAT (2.3 ± 1.0) than in MAT (4.4 ± 1.1) (*p* < 0.05).

**(3) Pain scores**

The overall pain score was significantly lower in the CMPAT group (2.7 ± 1.2) compared to the MAT group (4.1 ± 1.9) (*p* < 0.05) (Table 3; Figure 5).

In ages 6–49 years, CMPAT had lower pain scores (2.3 ± 1.3) than MAT (3.5 ± 2.0) (*p* < 0.05).

In ages ≥50 years, CMPAT also had significantly lower pain scores (3.2 ± 0.9) than MAT (4.6 ± 1.7) (*p* < 0.05).

Number of attempts positively correlated with pain scores (*R* = 0.812, *p* < 0.01).

**(4) Complications**

No significant difference in complications was found between CMPAT (3.3%) and MAT (8.3%) (*p* > 0.05).

The incidence of transient unilateral lower limb pain was 3.3% (2/60) in the CMPAT group and 8.3% (5/60) in the MAT group. However, the difference between groups was not statistically significant (*p* > 0.05).

### 3.3 Evaluation of lumbar puncture

No significant difference in complications was found between CMPAT and MAT groups (*p* > 0.05). The incidence of unilateral lower limb pain was 3.3% (2/60) in the CMPAT group and 8.3% (5/60) in the MAT group. No other complications were observed.

## 4 Discussion

Lumbar puncture (LP) is an essential neurological procedure enabling cerebrospinal fluid analysis and indirect intracranial pressure measurement, thereby aiding diagnosis of various neurological conditions (2). Therapeutically, LP can also facilitate CSF drainage using lumbar drains (4). However, conventional midline LP often fails, disrupting clinical workflows and causing stress for practitioners and patients alike (8). The paramedian approach was thus introduced to enhance access and avoid narrow interspinous spaces (6). But paramedian approach lacks standardization and exhibits variability across techniques (6, 14–17).

Previously, we developed an optimized computer-modified paramedian approach (CMPAT) using 3D modeling to maximize the target area and permissible errors, thereby improving LP success (18, 20). Here, we conducted a clinical study validating CMPAT versus conventional midline LP.

Though CMPAT was based on supine CT data, the association between the lamina edge and spinous process tip remains relatively constant despite flexed LP positioning (21). The lower lamina midpoint is the optimal target. Gradually increased cephalad tilt insertion angles can circumvent the lower lamina to access the enlarged interlaminar space (16). The lower spinous tip and posterior thoracolumbar fascia provide stable CMPAT landmarks (22), enabling avoidance of midline structures (23).

### 4.1 CMPAT reduces failure rates, especially in older adults

CMPAT significantly reduced overall failure rates compared to MAT (3.3% vs. 13.3%, *p* < 0.05) (Figure 5), especially in patients aged ≥50 years (0% vs. 17.6%, *p* < 0.05). Despite its long history, MAT struggles with high failure rates due to factors like age-related calcification and stenosis of interspinous spaces, improper positioning, and anatomical variations (6).

Failed LPs require more attempts, elevating procedural pain and risks of traumatic puncture (24). They also provoke anxiety in patients, families, and clinicians. CMPAT optimizes the target puncture area and exhibits versatility across age groups (Figure 1), substantially expanding the dural sac access window and permissible error range. This effect was more pronounced in elderly patients.

However, CMPAT failed in two severely obese patients, likely due to inadequate needle length given the longer path required. Successful puncture was achieved with MAT instead for these patients. Thus, CMPAT should be avoided in cases of severe obesity where needle manipulation may be hindered (25).

Though proficient skills are undoubtedly critical, innovative computational methodology may further aid conventional approaches by enhancing trajectory optimization for a wider, more heterogeneous patient population (26–28). This could increase probability of rapid successes especially among complex cases.

Consistently, our study proves CMPAT significantly reduces overall failure rates compared to conventional midline approach, aligning with the reported procedural success variability across published evidence (6, 9–11). The superiority is especially prominent in patients aged ≥50 years (0% vs. 17.6% failure rate), likely attributed to age-related stenosis.

### 4.2 CMPAT requires fewer attempts and creates less pain

CMPAT required significantly fewer puncture attempts than MAT overall (2.0 ± 1.2 vs. 3.5 ± 1.6, *p* < 0.05) (Figure 5), especially among patients aged ≥50 years (2.3 ± 1.0 vs. 4.4 ± 1.1, *p* < 0.05). This can be attributed to CMPAT’s optimized target area, reduced risk of puncture obstruction, and enhanced error tolerance range. Together, these facilitate successful punctures with fewer attempts.

Unlike other paramedian approach techniques, CMPAT uses the inferior spinous process tip as the bony landmark, and the posterior thoracolumbar fascia for layered positioning. This provides a relatively stable association between the needle path and key anatomical structures like the lamina, spinous process, interlaminar space, and dural sac.

CMPAT also yielded significantly lower pain scores than MAT (2.7 ± 1.2 vs. 4.1 ± 1.9, *p* < 0.05), which positively correlated with fewer attempts (*R* = 0.812, *p* < 0.01) (Figure 5).

### 4.3 CMPAT-associated pain may differ from MAT-associated pain, and recovery may be faster in CMPAT

**(1) Similarities between the CMPAT and MAT**

LP procedures can induce invasive pain characterized by sharp pain (resulting from skin puncture) and traumatic pain (caused by tissue damage from the needle). Aδ and C nerve fibers transmit pain signals from these regions (29). Moreover, puncture needles can irritate the nerve roots in the lumbar spine, leading primarily to radicular pain. In such cases, physicians may temporarily halt the procedure and change the needle direction to the opposite side. Inadequate and untimely modification of the puncture plan may lead to nerve root injuries and loss of neurological function (29, 30). Inflammation and tissue repair occur following LP, contributing to pain during the healing process. The duration of this pain is associated with the time required for healing, with longer healing times corresponding to prolonged pain duration (29, 31).

**(2) Differences between the CMPAT and MAT**

In contrast to the midline approach (MAT) which traverses the supraspinous and interspinous ligaments (32), the computer-modified paramedian approach (CMPAT) involves navigating the posterior layer of the thoracolumbar fascia and paraspinal musculature. Since the interspinous space is narrower in MAT, its error tolerance range is smaller compared to CMPAT. Thus, MAT risks damaging periosteal tissue and tendon/ligament insertions, which are densely innervated and prone to cause pain (33).

Our findings indicate CMPAT is associated with less pain. Despite sensory innervation of the posterior thoracolumbar fascia (34), CMPAT emphasizes anesthesia during puncture. By maximizing the error tolerance range, CMPAT minimizes periosteal damage and trauma to tendon/ligament attachments, thereby lowering pain scores.

Additionally, muscles exhibit quicker healing and recovery compared to tendons/ligaments (35). Since MAT involves penetrating ligaments and tendon insertions that heal slowly, pain duration may be more prolonged versus CMPAT.

### 4.4 The likelihood of nerve root injury may be lower in CMPAT

In this study, the incidence of unilateral lower limb pain was 3.3% after CMPAT-LP and 8.3% after MAT-LP, though not statistically significant. The CMPAT trajectory is obstructed from the ipsilateral nerve root by the articular process, making it difficult to damage (36). The contralateral nerve root is positioned remotely, also reducing the risk of injury. Thus, CMPAT may confer a lower likelihood of nerve root injury compared to MAT.

### 4.5 Comparison with the ultrasound-guided approach

**(1) Technical characteristics and advantages**

Computer-assisted techniques like CMPAT enable preoperative planning and simulation using 3D modeling and computational analysis (26, 37–39). This facilitates identifying optimal population-based and patient-specific needle trajectories to improve LP success rates while avoiding critical anatomical structures. A key advantage of CMPAT is the ability to preoperatively optimize the puncture path to enhance surgical accuracy and safety, while minimizing unnecessary needle insertion attempts (26). However, computer-assisted methods rely on CT imaging and lack real-time flexibility.

In contrast, ultrasound guidance offers dynamic and real-time needle visualization and navigation during LP procedures. This significantly enhances outcomes in challenging clinical scenarios like obesity and advanced age (7, 23). Nonetheless, ultrasound has limitations including acoustic shadowing from bony and ligamentous structures obscuring visualization of the dural sac target (40). Operator skill is also critical for effective ultrasound application. Precise target detection and trajectory planning can prove challenging, and 3D spatial perspective is restricted.

**(2) Applicability and complexity**

Computer assistance and ultrasound guidance have complementary strengths and limitations. Computer modeling optimizes pre-procedural planning, while ultrasound enables intra-procedural visualization. Merging these modalities can potentially harness their combined benefits for enhancing LP efficacy and safety (41). This is an area warranting future exploration.

**(3) Usage conditions**

For most general patient populations, the optimized CMPAT trajectory can be directly applied without needing patient-specific CT data or real-time ultrasound guidance. This study demonstrated its effectiveness under such conditions.

**(4) Merging CT and ultrasound**

In challenging cases like obesity or anatomy variations, merging CT and ultrasound could be valuable by combining their complementary strengths (42, 43). CT provides 3D anatomical details for pre-procedural planning, while ultrasound enables real-time visualization and dynamic needle guidance.

Fused CT-ultrasound imaging can integrate the global perspective from CT with live imaging from ultrasound (42, 43). This has strong potential to enhance LP success in difficult scenarios, overcoming limitations of either modality alone. Further research on CT-ultrasound fusion is warranted for such complex LPs.

### 4.6 Limitations

This single-center observational study lacked imaging data on participants’ lumbar degeneration. We were thus unable to characterize factors like interlaminar narrowing or osteoarthritic changes that could impact LP outcomes.

This study lacks direct comparison between CMPAT and existing paramedian techniques regarding performance metrics like failure rates, accuracy, pain levels across more scenarios. Further comparative research is valuable to thoroughly investigate their respective strengths, limitations, and suitability across diverse clinical situations and patient groups.

The modest sample size and short follow-up limit result in generalization. The advantages and risks of CMPAT warrant validation through larger multi-center randomized trials with extended follow-up.

Sub-group analysis by age was performed, but stratified analysis based on other parameters like gender, BMI, or lumbar pathology could provide further insights.

Patient-reported experience and satisfaction were not assessed. This important outcome should be included in future studies.

Longer-term monitoring of complications is needed.

## 5 Conclusion

Lumbar puncture is an essential neurological technique across many medical specialties. This study demonstrates that CMPAT reduces failure rates, lowers the number of attempts, and alleviates procedural pain compared to the conventional midline approach, without compromising safety.

CMPAT is a promising technique that optimizes puncture access through computational modeling. Our results support wider clinical implementation of CMPAT for improving LP outcomes and experience.

However, larger multi-center randomized trials with extended follow-up are warranted to further validate the efficacy and safety of CMPAT. Patient-reported experience should also be assessed in future studies.

## Data availability statement

The raw data supporting the conclusions of this article will be made available by the authors, without undue reservation.

## Ethics statement

The studies involving humans were approved by Ethics Committee of Fujian Medical University Union Hospital. The studies were conducted in accordance with the local legislation and institutional requirements. Written informed consent for participation in this study was provided by the participants’ legal guardians/next of kin. Written informed consent was obtained from the individual(s), and minor(s)’ legal guardian/next of kin, for the publication of any potentially identifiable images or data included in this article.

## Author contributions

Y-DZ: Conceptualization, Data curation, Funding acquisition, Investigation, Methodology, Project administration, Supervision, Validation, Visualization, Writing – original draft, Writing – review & editing. H-SX: Conceptualization, Data curation, Formal analysis, Investigation, Methodology, Project administration, Software, Supervision, Validation, Visualization, Writing – original draft, Writing – review & editing. JC: Data curation, Formal analysis, Methodology, Resources, Software, Validation, Visualization, Writing – original draft, Writing – review & editing. G-HW: Data curation, Formal analysis, Methodology, Resources, Software, Validation, Visualization, Writing – original draft, Writing – review & editing. J-FW: Conceptualization, Funding acquisition, Investigation, Methodology, Resources, Supervision, Visualization, Writing – original draft, Writing – review & editing. C-MC: Conceptualization, Formal analysis, Funding acquisition, Investigation, Methodology, Project administration, Supervision, Validation, Writing – original draft, Writing – review & editing.

## Supplementary material

SUPPLEMENTARY VIDEO 1 Showcases an animated demonstration of the puncture paths for CMPAT-LP and MAT-LP. The CMPAT technique, rooted in computational medicine, maximizes the target area on the dural sac and exhibits strong versatility. In comparison to MAT, the CMPAT puncture path significantly expands the target area on the dural sac and the range of error tolerance, facilitating successful punctures. The presence of the spinous process in the MAT technique narrows the puncture channel and increases the risk of periosteum damage. Abbreviations: CMPAT: computer-modified paramedian approach technique; MAT: midline approach technique; LP: Lumbar puncture [Video, MP4].

SUPPLEMENTARY VIDEO 2 Presents two illustrative procedures performed on patients who received CMPAT-LP. (a) Patient 1. A 72-year-old male who underwent L3-4 CMPAT-LP. (b) Patient 2. A 24-year-old male who underwent L4-5 CMPAT-LP. Abbreviations: CMPAT: computer-modified paramedian approach technique; LP: Lumbar puncture; PLTLF: the posterior layer of the thoracolumbar fascia [Video, MP4].
